# Synergistic Pain-Reducing Effects of *Bixa orellana* (Chronic^®^ and Chronic In^®^) and Cannabidiol-Rich *Cannabis sativa* Extracts in Experimental Pain Models

**DOI:** 10.3390/ph17121710

**Published:** 2024-12-18

**Authors:** Alicia de Melo Santos, Helison de Oliveira Carvalho, Danna Emanuelle Santos Gonçalves, Luciana Paes Gomes, Nayara Nilcia Dias Colares, Abrahão Victor Tavares de Lima Teixeira dos Santos, Adrielly Yasmin Sousa dos Santos, Thiago Afonso Teixeira, José Carlos Tavares Carvalho

**Affiliations:** 1 Laboratório de Pesquisa em Fármacos, Curso de Farmácia, Departamento de Ciências Biológicas e da Saúde, Universidade Federal do Amapá, Rod. Josmar Chaves Pinto, km 02—Jardim Marco Zero, Macapá—AP, Macapá 68903-419, AP, Brazil; aliciademeloo@hotmail.com (A.d.M.S.); helison_farma@hotmail.com (H.d.O.C.); danna.goncalves@gmail.com (D.E.S.G.); lucianapaees@gmail.com (L.P.G.); nayarandiascolares@gmail.com (N.N.D.C.); abrahaolima28@gmail.com (A.V.T.d.L.T.d.S.); adriellyyasmin18@gmail.com (A.Y.S.d.S.); thafonsoteixeira@gmail.com (T.A.T.); 2 Programa de Pós-Graduação em Inovação Farmacêutica, Departamento de Ciências Biológicas e da Saúde, Universidade Federal do Amapá, Macapá 68903-419, AP, Brazil; 3 Hospital Universitário, Universidade Federal do Amapá, R. do Estádio Zerão, s/n—Universidade, Macapá 68903-419, AP, Brazil

**Keywords:** *Cannabis sativa*, *Bixa orellana*, nanodispersion, granules, analgesia

## Abstract

**Background:** The present study aimed to evaluate the potential synergy between pharmaceutical formulations containing *Bixa orellana* L. (granulated—CHR OR and injectable nanodispersion—CHR IN) in conjunction with a cannabidiol (CBD)-rich extract of *Cannabis sativa* L. (CSE) on experimental pain models in Wistar rats. **Methods:** Chemical analysis was performed using gas chromatography (GC-MS). The pain tests employed were acetic acid-induced writhing (injection i.p. of 0.9% acetic acid), formalin (solution 1%), hot plate (55 ± 0.5 °C), and cold-water tail withdrawal tests. **Results:** Chemical analyses by chromatography confirmed that the oil from *B. orellana* is rich in δ-tocotrienol (72.0 ± 1.0%), while the oil from *Cannabis sativa* highlighted the presence of cannabidiol (CBD). The results from the experimental pain tests indicated that the combined administration of formulations containing *Bixa orellana* and *C. sativa*, such as the granulated CHR OR (400 mg/kg, orally) with CSE (40 mg/kg, orally) or the nanodispersion CHR IN (10 mg/kg, intramuscularly) with CSE (40 mg/kg, orally), demonstrated significant results (*p* < 0.001) in pain reduction. Although the formulations containing *Bixa orellana* extract showed statistical significance in the tests when used in isolation, their effects were inferior compared to the combined use with CSE or the isolated use of CSE. These findings suggest that combining formulations containing extracts of these plant species may represent a viable therapeutic option, considering the synergistic action in reducing pain under the experimental conditions employed. **Conclusions:** these results imply that combining the phytocomplexes present in *B. orellana* and *C. sativa* may be a promising approach for pain treatment.

## 1. Introduction

Pain is a distressing experience associated with actual or potential tissue damage, with sensory, emotional, cognitive, and social components [1]. Although pain generally plays an adaptive role, it can have adverse effects on function, social well-being, and psychological health, especially in the context of chronic pain, which affects approximately 20% of the population [2,3].

Pain treatments can take diverse pharmacological approaches depending on the type of pain experienced by the patient. For nociceptive pain, nonsteroidal anti-inflammatory drugs (NSAIDs), non-opioid analgesics, and opioids can be used. For neuropathic pain or central sensitization, tricyclic antidepressants and serotonin-norepinephrine reuptake inhibitors (SNRIs) are recommended options [3]. In response to the opioid epidemic and the risks associated with prescribing opioids for chronic pain management, alternative therapies have been introduced as viable treatment options, and the study of bioactive plant-derived compounds has garnered increasing interest [4].

*Bixa orellana* L., commonly known as “annatto” or “achiote,” is a small perennial tree native to the tropical rainforests of Central and South America. This species holds notable cultural significance in India, China, the Philippines, Brazil, and Guyana [5]. Additionally, the plant exhibits a wide range of medicinal properties. It is widely used to treat renal discomfort, diabetes mellitus, constipation, and hypertension, as well as an antibiotic, laxative, cardiotonic, anti-inflammatory, antipyretic, and analgesic [6,7]. These benefits are partly attributed to tocotrienols and geranylgeraniol in its composition [5].

The literature provides preclinical and clinical evidence supporting the analgesic effects attributed to tocotrienols, likely due to their anti-inflammatory properties [8]. Specifically, δ-tocotrienol, identified as the predominant isoform in *Bixa orellana* oil, has demonstrated significant efficacy in reducing inflammation and protecting cells from oxidative damage, as evidenced by studies [9,10].

*Cannabis sativa* L. is an herbaceous plant belonging to the *Cannabaceae* family characterized by its growth in tropical and subtropical regions [11]. The extensive medicinal history of *C. sativa* dates to ancient Chinese texts from 2900 BC, highlighting its ancestral use for pain relief [4]. Although *Cannabis sativa* contains a variety of cannabinoids, ∆9-tetrahydrocannabinol (∆_9_-THC) and cannabidiol (CBD) are the predominant components in terms of concentration [12].

CBD, a prominent non-psychoactive component of *Cannabis sativa*, has been the subject of extensive research due to its analgesic and anti-inflammatory properties. CBD demonstrates a limited affinity for the CB1 and CB2 receptors of the endocannabinoid system. Studies indicate that CBD may influence non-cannabinoid pathways, including serotonin 5-HT1A receptors, TRPV1, and GPR55, which play crucial roles in pain regulation [3,13].

The present study aimed to evaluate the potential synergy between pharmaceutical formulations containing *Bixa orellana* L. (granules—Chronic^®^ and injectable nanosuspension—Chronic In^®^) combined with CBD-rich *Cannabis sativa* L. extract in experimental pain models using Wistar rats.

## 2. Results and Discussion

### 2.1. Chemical Analysis of Bixa orellana and Cannabis sativa Oils

**Figure 1:**
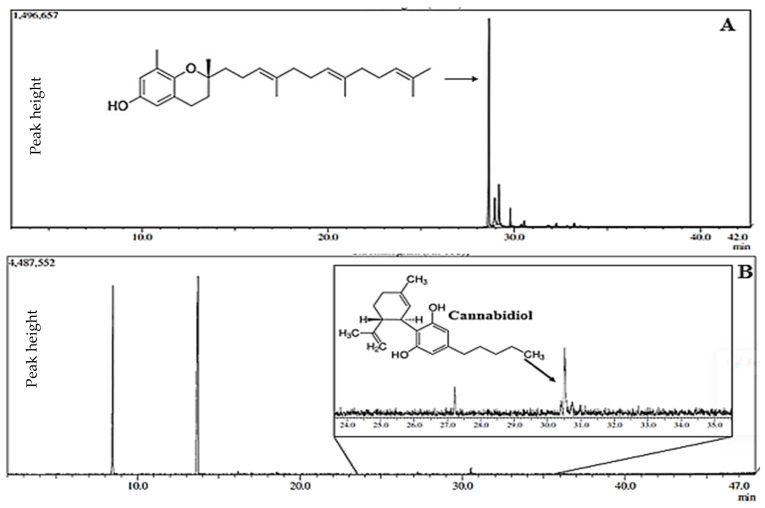
(**A**) Chromatographic profile of *Bixa orellana* oil with a major peak (area) corresponding to δ-tocotrienol (C_27_H_40_O_2_) at a retention time of 28.6 min. (**B**) Chromatographic profile of *Cannabis sativa* extract showing the retention time of cannabidiol at 30.54 min.

As analyzed by gas chromatography, the chromatogram (Figure 1) allowed for the integration of the peak corresponding to δ-tocotrienol (C_27_H_40_O_2_), with a retention time of 28.6 min. The average concentration of δ-tocotrienol was found to be 0.725 ± 0.062 mg/mL (72.0 ± 1.0%), calculated based on the equation of the line obtained from δ-tocotrienol at various concentrations. The coefficient of linearity (R^2^) was equal to 0.9973, with the regression equation given by ${y=8.30\times 10^{6}x-2.818\times 10}^{6}$.

The chemical results obtained for *Bixa orellana* oil are consistent with the literature, which reports δ-tocotrienol values ranging from 53% to 93% as the major component [14,15].

In the chemical analysis of *Cannabis sativa* oil via gas chromatography (Figure 1), the chromatographic peak of cannabidiol was identified at 30.54 min. The fragmentation profile was confirmed using the chromatograph’s NIST library. Other compounds were identified, including methyl esters of medium-chain fatty acids used to solubilize the extract, with the methyl ester of capric acid showing a retention time of 13.75 min.

Cannabidiol (CBD) has emerged as a promising substance due to its documented analgesic potential in various studies. This non-psychoactive compound, found in the *Cannabis sativa* plant, has been extensively investigated for its therapeutic properties, particularly in pain management [4].

### 2.2. Characterization of Injectable Nanodispersion (Chronic in^®^) of B. orellana

The nanoprecipitation method, adapted from Oliveira Carvalho et al. [16], was employed to prepare the nanodispersion containing *Bixa orellana* oil. No visual alterations indicating loss of stability were observed throughout the evaluation period. The formulation did not exhibit phase separation or the precipitation of components, demonstrating a visibly stable appearance with a slightly yellow color and a pH of 5.8 ± 0.01.

In this study, the nanoparticle size remained stable, with an average size of 53.15 ± 0.64 nm and a PDI of 0.574 ± 0.032 on the first day. On day 30, the particle size was 59.90 ± 3.63 nm with a PDI of 0.574 ± 0.032. The nanoformulation exhibited a Zeta potential of 19.86 ± 0.60 mV on day zero and 19.66 ± 1.45 mV on day 30 (Table 1).

The studies by Oliveira Carvalho et al. [11] and Mitchell et al. [17] have indicated that nanoformulations with particle sizes ranging from 50 to 200 nm significantly maintain stability, aligning with our findings. According to Pereira et al. [18], the reduction of nanoparticle size is crucial as it directly impacts the properties and stability of nanoemulsions. Smaller particles possess a larger surface area, leading to better dispersion and the reduced influence of repulsive forces, thereby preventing adjacent particles’ aggregation [19,20]. Additionally, Zeta potential, as highlighted by Zielinska et al. [21], is a crucial parameter for evaluating the physicochemical stability of nanoemulsions, with formulations above ±15 mV considered ideal.

### 2.3. Evaluation of Treatments in Pain Models

This study investigated the efficacy of subchronic treatment using a combination of *Cannabis sativa* extract with a high cannabidiol content and the nutraceuticals Chronic^®^ and Chronic In^®^, developed from the phytocomplex oil extract of *Bixa orellana* L., to alleviate pain in animals. We assessed the analgesic activity using four distinct methods: the acetic acid-induced writhing test, the formalin test, the hot plate test, and the cold-water tail withdrawal test.

The acetic acid-induced writhing technique is commonly employed to assess the efficacy of peripheral analgesic agents or visceral inflammatory pain management [22]. Acetic acid triggers an inflammatory response in the abdominal cavity, activating the nociceptor [23]. This model of visceral pain, induced by acetic acid nociception, is non-specific and peripheral, susceptible to modulation by various therapeutic agents, including anti-inflammatories, opioid analgesics, and other centrally acting compounds [23,24].

The pain-inducing action of acetic acid arises from its chemical stimulation in the peritoneal cavity, leading to the release of inflammatory mediators such as interleukins IL-1, IL-6, IL-8, tumor necrosis factor α (TNF-α), as well as chemokines, histamine, serotonin, and bradykinin. These substances promote the increased synthesis of lipoxygenase (LOX) and cyclooxygenase (COX) enzymes, resulting in the production of leukotrienes and prostaglandins, notably prostaglandin E2 (PGE2) and F2α (PGF2α) [24,25]. Additionally, pro-inflammatory cytokines such as IL-8, IL-1β, and TNF-α, released by macrophages and basophils, amplify the release of aspartate, glutamate, and other endogenous mediators that activate visceral nociceptive neurons [26].

Several recent studies have investigated the therapeutic potential of cannabidiol (CBD) in managing acetic acid-induced abdominal pain, consistently demonstrating its ability to significantly reduce abdominal writhing compared to placebo groups [13,22,24]. Neelakantan et al. [13] and Foss et al. [23] report that CBD decreases the production of various inflammatory markers, such as TNF-α, IL-6, and COX-2, through mechanisms that do not involve CB1 or CB2 cannabinoid receptors. Thus, the antinociceptive effects of CBD observed in the acetic acid-induced writhing test may be attributed to its potent anti-inflammatory properties.

Furthermore, earlier studies have corroborated the therapeutic efficacy of *Bixa orellana* in this same test. Aktary et al. [6] and Shilpi et al. [7] suggest that the antinociceptive activity observed in *Bixa orellana* extract may result from its ability to interfere with the synthesis and release of endogenous substances induced by acetic acid, such as prostacyclin (PGI_2_) and other prostanoids. Additionally, the desensitization of sensory C fibers may be involved in pain transmission.

**Figure 2:**
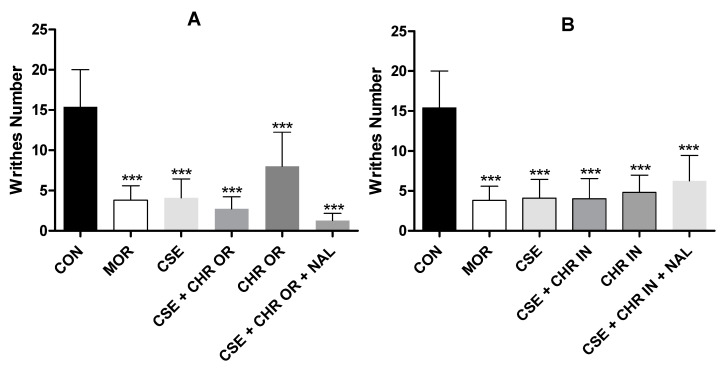
Effect of treatment with CSE (40 mg/kg) associated with Chronic^®^ (400 mg/kg) and CSE associated with Chronic In^®^ (10 mg/kg) in the acetic acid-induced abdominal writhing test. (**A**) represents the number of writhes in the CSE + CHR OR, CHR OR, and CSE + CHR OR + NAL groups. (**B**) represents the number of writhes in the CSE + CHR IN, CHR IN, and CSE + CHR IN + NAL groups. Results are presented as mean ± SD (n = 5). Differences between groups were analyzed by one-way analysis of variance (ANOVA), followed by Tukey’s test. *** indicates *p* < 0.001 vs. CON (control group).

Regarding the amount of abdominal writhing induced by acetic acid (Figure 2), a statistically significant reduction was observed in all experimental groups, with a *p*-value < 0.001. Notably, the groups subjected to the combined administration of CSE (40 mg/kg, oral) + CHR OR (400 mg/kg, oral), as well as those receiving this combination together with naloxone (2 mg/kg, intraperitoneal), showed results comparable to the standard drug (morphine).

Considering that the acetic acid-induced abdominal writhing test is a non-specific model and does not allow for the exact nociceptive pathways through which a drug may act to be determined, other procedures were employed to elucidate the possible mechanisms of antinociception [24]. The formalin test stands out from conventional pain models as it evaluates an animal’s response to continuous and moderate pain from tissue injury. It is considered more representative of clinical pain than tests that use short-duration mechanical or thermal stimuli [27].

Two distinct phases characterize the nociceptive response to formalin. The first phase, known as the neurogenic phase, occurs immediately after the formalin injection and lasts approximately 10 min. It is marked by releasing P substance, glutamate, bradykinin, and nitric oxide, which stimulate C-type nerve fibers and some Aδ fibers. The second phase, known as the inflammatory phase, begins 15–20 min after injection and can last for over 60 min, releasing inflammatory mediators such as histamine, serotonin, bradykinin, and prostaglandins [22,25,28]. Central-acting analgesics, such as opioids, inhibit both phases of the response, while peripherally acting anti-inflammatory drugs, such as nonsteroidal anti-inflammatory drugs (NSAIDs) and steroids (SAIDs), inhibit only the second phase [24].

**Figure 3:**
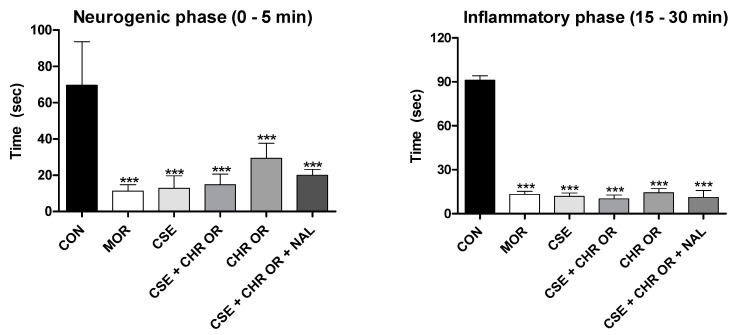
Formalin test. The rats (n = 5) were treated with placebo, morphine (10 mg/kg), CSE (40 mg/kg), CSE + CHR OR (400 mg/kg), and CSE + CHR OR + NAL (2 mg/kg). Nociceptive behaviors were recorded over time (seconds). Early phase (neurogenic phase: 0–5 min) and late phase (inflammatory phase: 15–30 min). Differences between groups were analyzed by one-way analysis of variance (ANOVA), followed by Tukey’s test. *** indicates *p* < 0.001 vs. CON (control group).

Figure 3 graphically presents the results obtained in this study for both test phases regarding the treatments with the orally administered nutraceutical Chronic^®^, either alone or in combination with *Cannabis sativa* extract rich in cannabidiol (CSE). In both the early and late phases, all treated groups showed significant differences from the control group, which received distilled water (4 mL/kg), with *p*-values < 0.001.

**Figure 4:**
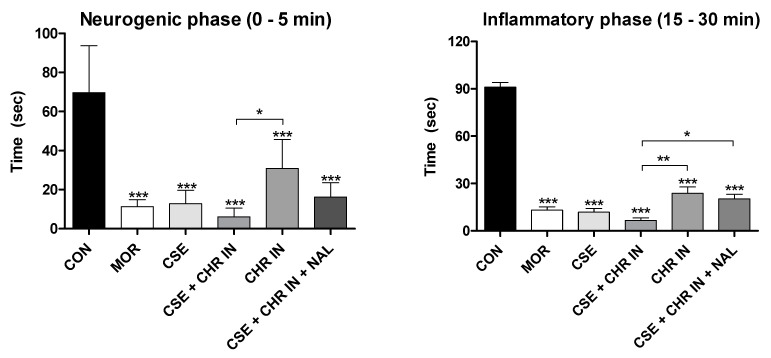
Formalin test. The rats (n = 5) were treated with placebo, morphine (10 mg/kg), CSE (40 mg/kg), CSE + CHR IN (10 mg/kg), and CSE + CHR IN + NAL (2 mg/kg). Nociceptive behaviors were recorded over time (seconds). Early phase (neurogenic phase: 0–5 min) and late phase (inflammatory phase: 15–30 min). Differences between groups were analyzed by one-way analysis of variance (ANOVA), followed by Tukey’s test. *, **, and *** indicate *p* < 0.05, *p* < 0.01, and *p* < 0.001, respectively, vs. CON (control group).

Regarding the results of the formalin test in the groups that received Chronic In^®^, administered intramuscularly, all groups showed significant differences compared to the control group (*p* < 0.001) in both the early and late phases (Figure 4). However, significant differences were observed between the experimental groups CSE + CHR IN and CHR IN alone in the early phase (*p* < 0.05) and the late phase (*p* < 0.01), with the group receiving the combination of treatments yielding the best results. This suggests that the combination of *Cannabis sativa* extract rich in cannabidiol may enhance the analgesic effects of *Bixa orellana*.

Additionally, in the late (inflammatory) phase, the combination of naloxone with CSE and CHR IN had a significantly negative impact (*p* < 0.05), increasing the nociceptive behavior of the animals. This effect was not reported when naloxone was combined with CSE and CHR OR, suggesting that the relevance of this interaction in the opioid pathway is still unclear. Therefore, further studies are necessary to elucidate this issue.

The findings of this study align with previous research highlighting the efficacy of CBD [22,24,29] and *Bixa orellana* [6] in this pain model. It is worth noting that CBD demonstrated a more pronounced influence in the inflammatory phase than in the neurogenic phase in all these studies.

Several studies corroborate the anti-inflammatory effects of CBD. Costa et al. [30] reported that, in a carrageenan-induced paw edema experiment, CBD significantly reduced tissue cyclooxygenase activity, plasma levels of prostaglandin E2, and the production of oxygen free radicals and nitric oxide. Atalay et al. [31] affirmed that CBD acts as an agonist of the PPARγ receptor, modulating inflammation by suppressing the expression of pro-inflammatory genes, including cyclooxygenase (COX2), and various pro-inflammatory mediators such as TNF-α, IL-1β, and IL-6. Additionally, Urits et al. [3] documented that, in combination with THC, even low concentrations of CBD can interact with CB2 receptors, inhibiting the inflammatory response, especially by suppressing mast cell degranulation and neutrophil migration to areas near pain centers. Therefore, the enhanced efficacy of CBD during the late phase of the formalin test could be attributed to its anti-inflammatory effects.

As for the action of *Bixa orellana* extract in the formalin test, its analgesic properties can also be associated with its anti-inflammatory characteristics [31]. As mentioned earlier, tocotrienols exhibit significant anti-inflammatory and antioxidant activity, reducing the secretion of critical pro-inflammatory mediators involved in pain sensitization and protecting against tissue injury [8,9,10]. Geranylgeraniol is another compound found in *B. orellana*, which demonstrates anti-inflammatory properties. Batista et al. [5] indicate that geranylgeraniol inhibits the expression of interleukin-1 receptor-associated kinase-1 (IRAK1) and tumor necrosis factor receptor-associated factor 6 (TRAF6), preventing excessive NF-κB activation in response to lipopolysaccharide-induced inflammation in THP-1 cells.

The hot plate and tail withdrawal tests are widely recognized as effective for evaluating central antinociceptive activity [6]. In this assay, the nociceptive experience is mediated by the activation of vanilloid receptors and the stimulation of C-type and Aδ-type nociceptive fibers [25]. Preliminary studies suggest that centrally acting drugs, such as NMDA receptor antagonists, opioids, and tricyclic antidepressants, show significant efficacy in this model. At the same time, anti-inflammatory agents like indomethacin, dexamethasone, and nimesulide do not exhibit relevant efficacy [28].

In the literature, there is some disagreement regarding the expected outcomes when assessing CBD in the hot plate test. While some studies report modest or no significant analgesic activity in this assay [13,23,32], casting doubt on the possibility of CBD's central analgesic action, other studies have found positive results for both CBD [33] and *Cannabis sativa* roots [25].

Regarding the analgesic effects of CBD in this test, Nascimento et al. [33] demonstrated that the selective CB1 antagonist AM251 can block CBD’s effect in the hot plate test. In contrast, the CB2 inverse agonist SCH 336 does not influence the antinociceptive effects of CBD, suggesting that the analgesic action of CBD in this test is mediated through the CB1 receptor rather than the CB2 receptor. Moreover, the antagonism of TRPV1 receptors amplified the antinociceptive effect of CBD. These findings indicate that CBD reduces nociception through FAAH inhibition, leading to increased levels of anandamide (AEA). Consequently, AEA may bind to both CB1 and TRPV1 receptors, which have opposing effects on pain modulation.

As for the antinociceptive effects of *Bixa orellana*, there is a notable gap in the literature, with few studies available. Aktary et al. [6] demonstrated that the methanolic Extract of *B. orellana* showed a latency time comparable to the control group in the hot plate test, suggesting a potential central analgesic action by this plant.

**Figure 5:**
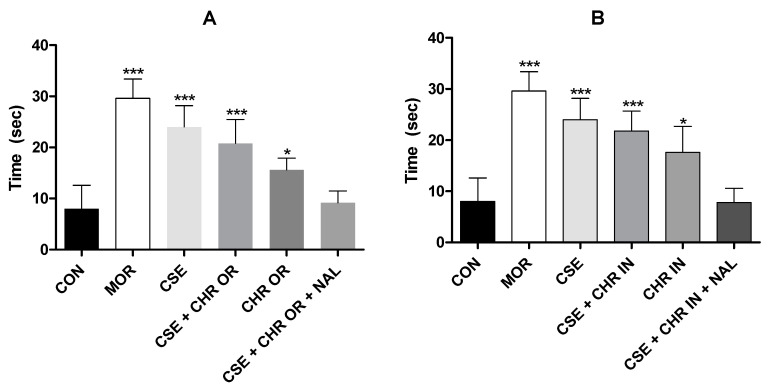
Effect of treatment with CSE (40 mg/kg) associated with Chronic^®^ (400 mg/kg) and CSE associated with Chronic In^®^ (10 mg/kg) in the hot plate test. (**A**) represents the latency time (seconds) in the CSE + CHR OR, CHR OR, and CSE + CHR OR + NAL groups. (**B**) represents the latency time (seconds) in the CSE + CHR IN, CHR IN, and CSE + CHR IN + NAL groups. Results are presented as mean ± SD (n = 5). Differences between groups were analyzed by one-way analysis of variance (ANOVA), followed by Tukey’s test. * and *** indicate *p* < 0.05 and *p* < 0.001, respectively, vs. CON (control group).

In our study, as illustrated in Figure 5, both the Group treated with CSE alone and the groups that received CSE in combination with Chronic^®^ or Chronic In^®^ exhibited statistically significant results (*p* < 0.001). The isolated use of CHR OR and CHR IN, although significant (*p* < 0.05), revealed inferior results compared to the other experimental groups, supporting the theory that *Bixa orellana* has a central analgesic effect, albeit less potent than that of CBD. It is important to note that the groups treated with naloxone, an opioid antagonist, displayed altered results, suggesting a potential interaction of CSE within the opioid pathway in this pain model. Some studies have already suggested a possible action of CBD on μ and δ opioid receptors for pain regulation [3,12], emphasizing further research to clarify this interaction.

As previously mentioned, the tail withdrawal test is widely recognized for assessing central antinociceptive activity. Unlike the hot plate test, where the endpoint involves a complex behavior (licking of the hind paw), nociceptive stimulation by temperature on the tail produces a simple nociceptive reflex response mediated by the spinal cord (tail withdrawal) [28]. According to studies [34,35], the cold-water tail withdrawal test is particularly suited for evaluating opioid-mediated antinociception. In this method, central nervous system depressants, such as antipsychotics or anxiolytics, as well as non-narcotic analgesics like aspirin and acetaminophen, are ineffective [35].

In the current literature, the results of the tail withdrawal test involving *Cannabis sativa*, particularly the phytocannabinoids CBD and THC, are conflicting. While specific studies fail to demonstrate therapeutic efficacy for CBD in this method, indicating that analgesic action is attributable solely to THC [36,37], other studies suggest analgesic effects of CBD, as documented by Nascimento et al. [33], who observed a significant reduction in thermal hyperalgesia in both acute and chronic CBD treatments. Regarding the action of *Bixa orellana* in this context, Aktary et al. [6] identified significant neuropharmacological activity in a dose-dependent manner comparable to standard drugs.

**Figure 6:**
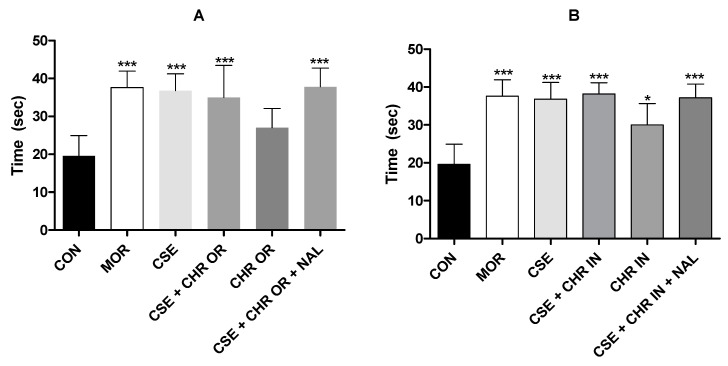
Effect of treatment with CSE (40 mg/kg) associated with Chronic (400 mg/kg) and CSE associated with Chronic In (10 mg/kg) in the cold-water tail withdrawal test. (**A**) represents the tail withdrawal time (seconds) in the CSE + CHR OR, CHR OR, and CSE + CHR OR + NAL groups. (**B**) represents the tail withdrawal time (seconds) in the CSE + CHR IN, CHR IN, and CSE + CHR IN + NAL groups. Results are presented as mean ± SD (n = 5). Differences between groups were analyzed by one-way analysis of variance (ANOVA), followed by Tukey’s test. * and *** indicate *p* < 0.05 and *p* < 0.001, respectively, vs. CON (control group).

Figure 6 presents the results of the cold-water tail immersion test, which measures the time required for tail withdrawal. The groups treated with CSE (40 mg/kg V.O.) + CHR OR (400 mg/kg V.O.) and CSE (40 mg/kg V.O.) + CHR IN (10 mg/kg V.O.), as well as their combinations with naloxone (2 mg/kg intraperitoneally), demonstrated a significant increase in response time. This increase is evidenced by *p* < 0.001 compared to the control group and is comparable to both the positive control group (MOR) and the group treated with CSE alone, with *p* < 0.001.

The group that received CHR IN alone showed statistical significance (*p* < 0.05), although inferior to those that received the combinations. However, the group that received CHR OR did not show significant differences compared to the control group. These results suggest that combining CSE with CHR OR or CHR IN enhances analgesic effects, while naloxone does not appear to interfere with these effects significantly.

## 3. Materials and Methods

### 3.1. Acquisition of Cannabis sativa Extract and Chemical Analysis by G.C.

The *Cannabis sativa* extract was obtained from Zion Medpharma (São Paulo, SP, Brazil), with an extract concentration of 200 mg/mL, containing 50 mg/mL of CBD and <2 mg/mL of THC. The gas chromatography (GC 2010, MS2010 Plus mass detector, Shimadzu corporation, Kyoto, Japan) analysis conditions were capillary column RTX-5 (30 m × 0.25 mm ID × 0.25 µm) and helium as the carrier gas, with a 14 mL/min flow rate. The oven temperature was programmed to remain at 60 °C for 1 min, then increase by 5 °C/min up to 300 °C. The injector temperature was set to 280 °C, and the detector temperature to 250 °C. A 1 µL injection was performed in split mode at a 10:1 ratio. The fragmentation spectra of the compounds were evaluated and identified using the NIST library of the equipment.

### 3.2. Acquisition of B. orellana Oil and Chemical Analysis

The *B. orellana* oil (Chronic^®^) was provided by Ages Bioactive Compounds, batch 0012/2022. For the chemical analysis, δ-tocotrienol quantification was performed. A stock solution of δ-tocotrienol in hexane (Sigma-Aldrich, MKCF5755, G.C. grade, Steinheim, Höxter, Germany) was prepared at 10 mg/mL concentration. From this stock solution, additional solutions were prepared to construct a calibration curve with the following concentrations: 2.5 mg/mL, 1.0 mg/mL, 0.5 mg/mL, 0.25 mg/mL, and 0.1 mg/mL. The analyses were conducted using gas chromatography-mass spectrometry (GC-MS, Shimadzu corporation, Kyoto, Japan), following the previously described methodology [38].

### 3.3. Obtention of the Granules of Bixa orellana (Chronic^®^)

The granules of *Bixa orellana* (Chronic^®^) were sourced from Ages Bioactive Compounds Co. (São Paulo, SP, Brazil). The extraction technique follows a standardized process protected by a patent.

### 3.4. Obtention of Polymeric Nanoparticles Containing Bixa orellana Oil (Chronic in^®^)

The nanoprecipitation method, as adopted by de Oliveira Carvalho et al. [16], was used to create a polymeric nanoparticle system containing *Bixa orellana* oil. A formulation of 1% purified *B. orellana* oil, 2% polyethylene glycol 4000 (PEG 4000), 2% Tween 80, 25% absolute ethanol, and 70% injectable-grade water was used to produce a final volume of 20 mL of nanosuspension (Chronic In^®^).

First, the organic phase was prepared by solubilizing *B. orellana* oil and PEG 4000 in absolute ethanol and stirring the mixture with a magnetic stirrer (GT-AMB10L—Global Equipment, Minas Gerais, Brazil) at 800 rpsgm for 20 min. Simultaneously, the aqueous phase was prepared by solubilizing Tween 80 in water and stirring for 20 min. The organic phase was poured into the aqueous phase under agitation using a mechanical stirrer (Fisatom, São Paulo, Brazil) for 20 min. After preparation, the resulting nanosuspension was stored in injectable ampoules and subjected to characterization.

### 3.5. Characterization of B. orellana Nanosuspension (Chronic in^®^)

For stability purposes, nanosuspension was characterized on the day of preparation and after 7 and 30 days. Visual appearance, phase separation, and sedimentation were macroscopically evaluated. Zeta potential, polydispersity index (PDI), and particle size were assessed in triplicate using a Zeta-Sizer (Malvern Pan-Analytics, Nottingham, UK), following the methodology described by Borges et al. [39].

### 3.6. Study Design and Ethical Considerations

This study was a randomized, controlled, non-clinical trial utilizing quantitative methods. The research was conducted at the Drug Research Laboratory located in the Health Sciences building of the Federal University of Amapá (UNIFAP), situated at Rodovia Juscelino Kubitscheck, Km-02, s/n, Zerão, Macapá, Amapá, Brazil.

As the study involved the use of animals, all procedures were conducted following the recommendations of the Universal Declaration of Animal Rights, the Brazilian College of Animal Experimentation (COBEA), and the resolutions of the Federal Council of Veterinary Medicine, as well as other applicable laws governing ethical practices for experimental research involving animals.

The study protocol was submitted to the Animal Research Ethics Committee (CEUA) of the Federal University of Amapá (UNIFAP) for review and was approved under protocol number 02/2022.

### 3.7. Animals Used

A total of 45 female Wistar rats were used in this study. The rats were obtained from the Multidisciplinary Center for Biological Research in Laboratory Animal Science (CEMIB)—University of Campinas (UNICAMP) at 21 days of age. The animals were acclimatized in groups of five per polyethylene cage, maintained under controlled temperature conditions (21 ± 1 °C), with free access to food and potable water, a 12-h light/dark cycle, and sawdust bedding changes, at the Drug Research Laboratory of the Federal University of Amapá.

### 3.8. Experimental Design

The present study utilized nine experimental groups comprising five Wistar strain Rattus norvegicus. For 28 days, seven of these groups were subjected to subchronic treatment administered daily.

The Negative Control Group (CON) received distilled water orally at 4 mL/kg. The Chronic^®^ Group (CHR OR) was treated with Chronic^®^ orally at 400 mg/kg. The Chronic In^®^ Group (CHR IN) received Chronic In^®^ via intramuscular injection (I.M.) at 10 mg/kg.

Other groups received combinations of treatments: the *Cannabis sativa* extract Group + Chronic^®^ (CSE + CHR OR) was treated with CSE orally at a dosage of 40 mg/kg and Chronic^®^ orally at a dosage of 400 mg/kg; the *Cannabis sativa* extract + Chronic In^®^ Group (CSE + CHR IN) was treated with CSE orally at a dosage of 40 mg/kg and Chronic In^®^ I.M. at a dosage of 10 mg/kg. The *Cannabis sativa* extract + Chronic^®^ + Naloxone Group (CSE + CHR OR + NAL) was treated with CSE orally at a dosage of 40 mg/kg, Chronic^®^ at a dosage of 400 mg/kg, and intraperitoneal Naloxone (I.P.) at a dosage of 2 mg/kg. Lastly, the *Cannabis sativa* extract + Chronic In^®^ + Naloxone Group (CSE + CHR IN + NAL) received CSE orally at a dosage of 40 mg/kg, Chronic In^®^ I.M. at a dosage of 10 mg/kg, and intraperitoneal Naloxone at a dosage of 2 mg/kg.

Two of the nine experimental groups received treatment only one hour before each experiment: the Positive Control Group (MOR), treated with morphine orally at a dosage of 10 mg/kg, and the *Cannabis sativa* extract group (CSE), treated with CSE orally at a dosage of 40 mg/kg.

### 3.9. Formalin Test

This test was conducted according to the methodology proposed by Dubuisson and Dennis [40]. A total of 0.02 mL of a 1% formalin solution was administered intraplantarly in the right hind paws of the animals. Each animal's time spent licking its paw was recorded in two phases: the first from 0 to 5 min and the second from 20 to 30 min. The groups were subjected to daily treatments for 28 days. The test was performed on the 28th day, 60 min after administering treatments. The MOR and CSE groups were exceptions, receiving treatment only on the day of the experiment and a 60-min interval before the test.

### 3.10. Hot Plate Test

The animals were placed on a hot plate at a fixed temperature (55 ± 0.5 °C). The latency to heat was assessed by measuring the time the animal took to withdraw its hind paw from the hot plate and lick it. The cut-off time was thirty seconds [41]. The groups were subjected to daily treatments for 28 days. The test was conducted on the 28th day, with measurements initiated 60 min after administering treatments. The MOR and CSE groups were exceptions, receiving treatment only on the day of the experiment and a 60-min interval before the measurements.

### 3.11. Cold-Water Tail Withdrawal Test

Tail withdrawal tests in cold water were performed according to the method described by Sharma et al. [42]. The distal portion of each rat’s tail was immersed in cold water (10 ± 1 °C). Baseline latency for tail movement (tail withdrawal response) or signs of struggling were observed. The cut-off time was set at 50 s. The groups were subjected to daily treatments for 28 days. The test was conducted on the 28th day, 60 min after administering treatments. The MOR and CSE groups were exceptions, receiving treatment only on the day of the experiment and a 60-min interval before the test.

### 3.12. Acetic Acid-Induced Abdominal Writhing

An intraperitoneal injection of 0.9% acetic acid (volume 0.1 mL/10 g) diluted in saline (0.9%) was administered. After the administration of acetic acid, the rats were placed in an acrylic box and observed for counting abdominal writhing from the fifth to the twentieth minute (adapted from Collier et al. [43]). The groups were subjected to daily treatments for 28 days. The test was conducted on the 28th day, 60 min after administering treatments. The MOR and CSE groups were exceptions, receiving treatment only on the day of the experiment and a 60-min interval before the test.

### 3.13. Statistical Analysis

All experimental quantitative results were expressed as mean ± standard deviation for statistical analysis. Tukey’s multiple comparisons test followed a one-way analysis of variance (ANOVA). Results with significance levels of *p* < 0.05 were considered statistically significant. The statistical software used for analysis was GraphPad Instat and Prism (version 5.03).

## 4. Conclusions

The simultaneous administration of formulations containing *Bixa orellana* and *C. sativa*, such as the granules CHR OR (400 mg/kg, orally) with CSE (40 mg/kg, orally) or the nanodispersion CHR In (10 mg/kg, intramuscularly) with CSE (40 mg/kg, orally), yielded significant and promising results in reducing pain in Wistar rat pain models, suggesting a potential synergy between the formulations. While the *Bixa orellana* extract showed statistical significance when used alone, its effects were less pronounced than the combined use with CSE or the isolated use of CSE. These findings imply that combining formulations containing extracts of these plant species may offer a viable therapeutic option, as it enhances pain reduction under the experimental conditions.

## 5. Patents

The work is part of patent BR 10 2023 006029 3 A2 “Composition, the process for obtaining polymeric nanoparticles containing *Bixa orellana* oil standardized in total tocotrienols, the process of the polar microparticulate system or natural microcluster and its uses”, deposited at the National Institute of Industrial Property—Brazil.

## Figures and Tables

**Table 1:** Analysis of Zeta potential (mV), average size (nm), and polydispersity index (PDI) of the nanodispersion (Chronic In^®^), analyzed at 1 and 30 days after the NBO was obtained, presenting mean and standard deviation (n = 3).

	P. Zeta (mV)	Size (nm)	PDI	pH
**Day 1**	19.86 ± 0.60	53.15 ± 0.64	0.533 ± 0.008	5.8 ± 0.01
**Day 30**	19.66 ± 1.45	59.90 ± 3.63	0.594 ± 0.014	5.8 ± 0.01

## Data Availability

The original contributions presented in the study are included in the article, further inquiries can be directed to the corresponding author.
